# Dynamics of Specific cfDNA Fragments in the Plasma of Full Marathon Participants

**DOI:** 10.3390/genes12050676

**Published:** 2021-04-30

**Authors:** Takehito Sugasawa, Shin-ichiro Fujita, Tomoaki Kuji, Noriyo Ishibashi, Kenshirou Tamai, Yasushi Kawakami, Kazuhiro Takekoshi

**Affiliations:** 1 Laboratory of Laboratory-Sports Medicine, Division of Clinical Medicine, Faculty of Medicine, University of Tsukuba, 1-1-1 Tennodai, Tsukuba 305-8577, Ibaraki, Japan; take0716@krf.biglobe.ne.jp (T.S.); shin.ichiro.fujita.03@gmail.com (S.-i.F.); y-kawa@md.tsukuba.ac.jp (Y.K.); 2 Doctoral Program in Sports Medicine, Graduate School of Comprehensive Human Sciences, University of Tsukuba, 1-1-1 Tennodai, Tsukuba 305-8577, Ibaraki, Japan; s1930394@s.tsukuba.ac.jp; 3 Research and Development Division, Blue Industries Inc., ArcaCentral Bldg 14F, 1-1-1 Kinshi, Sumida, Tokyo 130-0013, Japan; 4 Tsukuba i-Laboratory LLP, 2-1-17 Amakubo, Tsukuba 305-0005, Ibaraki, Japan; ishibashi@tsukuba-i-lab.com (N.I.); tamai@tsukuba-i-lab.com (K.T.)

**Keywords:** cfDNA, next-generation sequencing, full marathon, exercise, physiology

## Abstract

Plasma cell-free DNA (cfDNA) is frequently analyzed using liquid biopsy to investigate cancer markers. We hypothesized that this concept might be applicable in exercise physiology. Here, we aimed to identify specific cfDNA (spcfDNA) sequences in the plasma of healthy humans using next-generation sequencing (NGS) and clearly define the dynamics regarding spcfDNA-fragment levels upon extreme exercises, such as running a full marathon. NGS analysis was performed using cfDNA of pooled plasma collected from healthy participants. We confirmed that the TaqMan-qPCR assay had high sensitivity and found that the spcfDNA sequence abundance was 16,600-fold higher than that in a normal genomic region. We then used the TaqMan-qPCR assay to investigate the dynamics of spcfDNA-fragment levels upon running a full marathon. The spcfDNA fragment levels were significantly increased post-marathon. Furthermore, spcfDNA fragment levels were strongly correlated with white blood cell and plasma myoglobin concentrations. These results suggest the spcfDNA fragments identified in this study were highly sensitive as markers of extreme physical stress. The findings of this study may provide new insights into exercise physiology and genome biology in humans.

## 1. Introduction

In recent years, many studies using plasma cell-free DNA (cfDNA) have been conducted in cancer research and are associated with the concept of liquid biopsy being a minimally invasive method. These studies are based on the physiological phenomenon of mutated DNA fragments of dead cancer cells leaking into the blood and remaining in the plasma. Therefore, these fragments can be detected as biomarkers of cancer outbreak and progression in patients with cancer [1,2,3].

For example, in a study of non-small cell lung cancer that included a large number of patients worldwide, it was found that quantifying the abundance of circulating tumor DNA (ctDNA) present in plasma cfDNA enables the evaluation of early diagnosis, the efficacy of molecular target drugs, and drug resistance [3]. Conventionally, cancer diagnosis and malignant degeneration evaluation have been performed using tissue biopsy. However, in recent years, plasma cfDNA has also been used and allows cancer parameters to be evaluated by employing minimally invasive methods without the need for tissue biopsy. Therefore, this method is considered a promising approach for the medical examination of cancers in the future [4].

Based on the findings of the cancer studies mentioned above, we conceived the possibility of applying plasma cfDNA in exercise physiology, as it is well known that excessive oxidative stress occurs during severe exercise [5,6], which induces cell death [7,8,9]. Accordingly, we inferred that oxidative stress caused by extreme exercise would induce cell death in the tissues of an individual, which, in turn, would enable the leakage of genomic DNA into the blood. In support of this hypothesis, previous studies have reported that severe exercise increases the absolute amounts of plasma cfDNA, suggesting that cfDNA may become a physical stress marker [10,11,12]. However, these previous studies are limited because they only quantified the absolute cfDNA amount, and there are no reports on sequence information regarding cfDNA. Genomic DNA exists as a nucleosome, with various proteins bound to it. Therefore, it can be inferred that depending on the binding site of the protein, some DNA regions are likely less susceptible to degradation by deoxyribonuclease (DNase). Therefore, it is important to clarify whether specific sequences tend to persist due to extreme exercise.

In the current study, we aimed to identify specific cfDNA (spcfDNA) sequences that may remain in the plasma by evaluating samples from healthy individuals using next-generation sequencing (NGS). We also aimed to clarify the dynamics regarding scfDNA-fragment levels upon extreme exercise by evaluating runners before and at various time points after completing a full marathon. By clarifying these parameters, we expect to obtain new insights into exercise physiology and genome biology.

## 2. Materials and Methods

### 2.1. Ethical Approval and Study Overview

**Figure 1:**
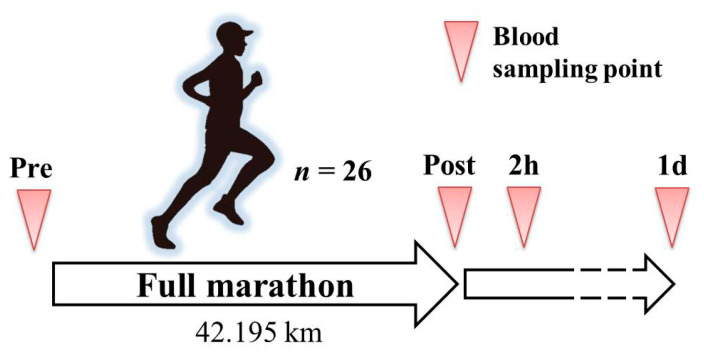
Schematic overview of the experimental protocol. Pre, immediately before the full marathon; ost, immediately after the full marathon; 2 h, two hours post full marathon; and 1 d, 1 day post full marathon.

This study was approved by the Ethical Committee of the Faculty of Medicine at the University of Tsukuba in accordance with the Declaration of Helsinki (approval number: 274). Before performing the experiments, all participants received an explanation and documents describing the purpose of the study, its design details, and potential safety issues, and each provided informed consent. An overview of the experimental protocol is shown in Figure 1. The participants enrolled in the current study, including their analyzed blood samples, were the same as those reported in our previously published online article [13] that focused on the fragment size and concentration of total cfDNA in the plasma. However, the current study presents additional new findings that are not included in the previous article, particularly those pertaining to the NGS analysis.

### 2.2. Study Participants

Twenty-six healthy males who perform aerobic exercises at least twice a week were recruited through a public notice. The enrolled participants planned to participate in the 38th Tsukuba Marathon, a full marathon sport event in Tsukuba City, Ibaraki Prefecture, Japan. The average age, height, and body weight (±standard deviation) of the participants were 25.2 (±7.3) years, 172.0 (±5.3) cm, and 64.9 (±6.9) kg, respectively. The participants were instructed not to consume alcohol, get a sufficient amount of sleep, and avoid binge eating before the full marathon. On the day of the full marathon, the participants freely performed warm-up exercises and drank water. All subjects finished the marathon, so there were no excluded subjects. The mean finish time ± standard deviation was 4:10:59 ± 0:59:03 (hours: minutes: seconds).

### 2.3. Blood Sample Collection

The outside air conditions on the day of the full marathon consisited of a temperature of 12.7 °C with a relative humidity level of 60.6%. Blood samples of the participants were collected into EDTA blood collection tubes at four time points, immediately before the marathon (Pre; also before warm-up), immediately after the marathon (Post), two hours after the marathon (2 h), and 1 day after the marathon (1 d). The participants were instructed to drink only water between the Post and 2 h collection points. The collected blood samples were centrifuged at 3000 rpm for 15 min at 4 °C. Aliquots of the plasma were then dispensed into 1.5 mL microtubes and stored at −80 °C until further analysis.

### 2.4. Measurement of General Stress Markers in the Blood Samples

Analysis of the blood samples was outsourced to a local clinical laboratory (Tsukuba i-Laboratory, Tsukuba, Ibaraki, Japan). The examination parameters in the hematological or biochemical examination were the number of white blood cells (WBCs), plasma myoglobin (MG) concentration, and enzyme activities of plasma creatine kinase (CK).

### 2.5. Extraction of cfDNA from Pooled Plasma Samples

Ninety microliters of individual plasma samples were pooled at each time point, and the pooled plasma (2.07 mL) was processed. Briefly, cfDNA in the pooled plasma samples was extracted using a NucleoSnap cfDNA Kit (Takara Bio, Shiga, Japan), according to the manufacturer’s instructions. Concentrations and size distributions of the cfDNA to perfume pre-preparation for subsequent analyses were measured using an Agilent Bioanalyzer (Agilent Technologies, Santa Clara, CA, USA) and an Agilent High Sensitivity DNA Kit (Agilent Technologies), according to the manufacturer’s instructions. The extracted cfDNA was stored at −20 °C until further analysis.

### 2.6. Extraction of cfDNA from Individual Plasma Samples

Using 200 μL of individual plasma samples at each time point, cfDNA was extracted using a NucleoSpin cfDNA XS Kit (Takara Bio), according to the manufacturer’s instructions. The final elution volume was 30 μL. Because the plasma cfDNA concentrations were very low and the concentrations could not be measured using a spectrophotometer, we used undiluted plasma-cfDNA samples for subsequent analysis. cfDNA was stored at −20 °C until further analysis.

### 2.7. Library Preparations for NGS

The NGS library was prepared using 3 ng of pooled plasma cfDNA from the “Pre” (healthy) time point and a SMARTer ThruPLEX Plasma-seq Kit (Takara Bio), according to the manufacturer’s instructions. The concentrations and fragment sizes of the libraries were measured using an Agilent High Sensitivity DNA Kit, according to the manufacturer’s instructions. The libraries were stored at −20 °C until further analysis.

### 2.8. NGS Analysis

The plasma cfDNA libraries were pooled and the concentrations adjusted to 2 nM. The pooled libraries were then diluted to 1.8 pM for the denaturation step. NGS was performed using a NextSeq 500 System (Illumina, San Diego, CA, USA) and NextSeq 500/550 v2.5 (75 Cycles) Kits (Illumina). The sequencing conditions were paired-end reads of 36 bases. After the sequencing run, a quality score over 30 was confirmed for 89.63% of all reads, indicating the success of the run. The read number was 205 million paired-end reads.

### 2.9. Bioinformatics Analyses

Bioinformatics analyses were performed using the CLC Genomics Workbench 20.0.3 software (QIAGEN, Hilden, Germany). FastQ files obtained from NGS were imported to the software, and poor-quality reads were trimmed or excluded using the program “Trim Reads” on the default settings. The trimmed reads were subjected to analysis using the “Map Read to Reference” program available on Genome Reference Consortium Human Build 37 (GRCh37; hg19). To identify plasma spcfDNA sequences, the “Transcription Factor ChIP-Seq” program was alternately used as a peak call using the default settings was alternatively used for mapping the data. We also evaluated the mapped data to visualize the genome regions mapped to spcfDNA sequences and normal sequences (glyceraldehyde 3-phosphate dehydrogenase; GAPDH cfDNA). A pre-sample BED file of pre-sample peaks was exported from the CLC Genomics Workbench software and added to the GAPDH region (chr12:6,645,100–6,645,500). To quantify the alignments from the BAM file with the overlap region in a BED file, a bedtools multicov function (version 2.30.0) was performed at the default setting using a pre-sample BED file against a position-sorted and indexed BAM file of the pre-sample using SAMtools (version 1.7). The values were expressed as transcripts per million (TPM) in python (version 3.8.5).

### 2.10. TaqMan-qPCR Assay

The TaqMan-qPCR primers and probe for the spcfDNA sequences (spcfDNA-1) identified by bioinformatics analysis and those for the genomic GAPDH (gGAPDH) region were designed using the Primer-BLAST (National Library of Medicine, Bethesda, MD, USA) web tool. The primers and probe as a double quencher system were synthesized by Integrated DNA Technologies (Coralville, IA, USA), and the sequences are shown in Table 1. The 1st TaqMan-qPCR assay was performed as duplicate measurements to quantify the spcfDNA-1 and GAPDH fragments present in the individual plasma-cfDNAs at the Pre time point (healthy) using PrimeTime Gene Expression Master Mix (Integrated DNA Technologies, Coralville, IA, USA) with the PCR primers and TaqMan probe on a QuantStudio 5 Real-Time PCR System (Thermo Fisher Scientific, Waltham, MA, USA). The template in a 2 μL volume, 200 nM of each primer, and 100 nM probe were included in a total reaction volume of 10 μL per well. Negative-control wells were also prepared, instead of templates, using distilled water (DW) in the assays with no amplification. The threshold cycle (CT) values obtained were converted to relative quantification values using the 2^−ΔΔCt^ method. The spcfDNA-1 fragments were also quantified at each time point on individual plasma-cfDNAs as absolute quantification using the same TaqMan-qPCR assay. Genomic DNA of the human embryonic fibroblast cell line JCRB 1006.7 (JCRB Cell Bank; original developers: Kouchi and Namba) was used to construct the standard curve for absolute quantification. The R^2^ of the standard curves was greater than 0.99.

### 2.11. Statistics

All data without the date on bioinformatics analyses (Section 2.9) were statistically analyzed using GraphPad Prism version 7.04 software (GraphPad, San Diego, CA, USA). To evaluate the values of each group, we first performed the Shapiro–Wilk normality test to verify distribution normality. We then performed non-parametric testing between all the groups. To test for differences between two groups, Mann–Whitney U tests were performed. For testing among the four groups, Kruskal–Wallis H tests (one-way ANOVA of ranks) were performed followed by a two-stage Benjamini, Krieger, and Yekutieli False Discovery Rate (FDR) procedure, which was run as a post hoc test with a defined pre-value as a control. For correlation analysis, we first converted the values for spcfDNA-1, WBC, MG, and CK to a common logarithm as log_10_. The correlation analyses were then performed between spcfDNA-1 and the WBC, MG, and CK values for all time points. Statistical significance was set at *p* < 0.05. In the graphs, the y-axes are displayed on normal or logarithmic scales.

## 3. Results

### 3.1. Confirmation of cfDNA Concentrations and Fragment Sizes in Pooled Plasma

**Figure 2:**
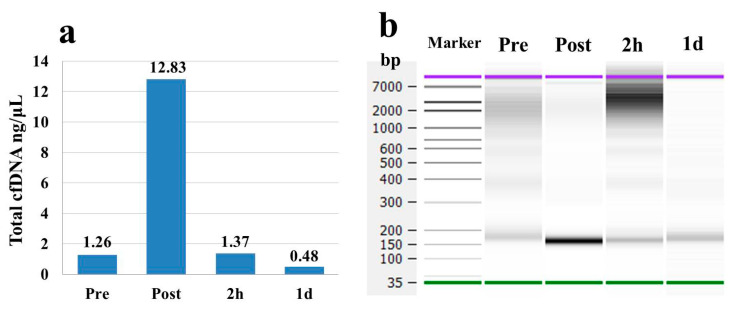
Total cfDNA concentrations and size distributions in pooled plasma of 26 participants. (**a**) Total cfDNA concentration measured using an Agilent Bioanalyzer. (**b**) Size distributions of cfDNA analyzed by gel electrophoresis in the Agilent Bioanalyzer.

Total cfDNA concentrations in the pooled plasma of 26 participants increased approximately 12-fold at the Post marathon time point compared to the Pre marathon time point (Figure 2a). The concentrations subsequently returned to baseline values at 2 h and then decreased at 1 d (Figure 2a). Meanwhile, the cfDNA fragments approximately 150 bp and 2–7 kb in size in the gel electrophoresis increased in brightness at Post and 2 h time points. At the 1 d time point, the brightness of short fragments approximately 150–200 bp returned to levels observed at the Pre time point (Figure 2b). These results suggested that concentrations of the total cfDNA fluctuated significantly as a result of the full marathon. Additionally, the cfDNA at Pre time point was decided to be suitable for preparing the NGS library.

### 3.2. spcfDNA Sequences Were Identified in the Pre Pooled Plasma cfDNA

**Figure 3:**
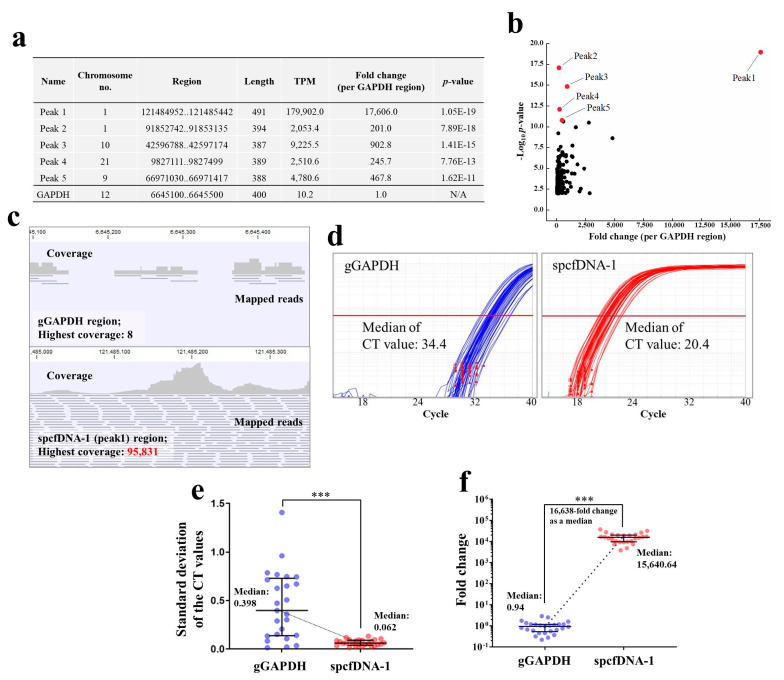
Identification of the spcfDNA region and evaluation of individual abundance. (**a**) Table of significant peaks of top 5 identified by bioinformatics analyses. Abbreviations N/A: not applicable. (**b**) Dot plot of −Log_10_ *p*-value and fold change (per GAPDH region). The top 5 peaks in (**a**) are colored in red dots. (**c**) Visualizations of the coverage and mapped reads on the control gGAPDH region and spcfDNA-1 region were identified in this study using pooled plasma cfDNA. (**d**) Individual TaqMan-qPCR amplification curves (*n* = 26) for gGAPDH and spcfDNA-1 fragments with median CT values indicated. The results are the average of duplicate measurements. (**e**) SD calculated from the individual CT values of data on (**d**). (**f**) Individual Taq-Man-qPCR quantification values for gGAPDH and spcfDNA-1 fragments. The individual data for *n* = 26 samples in each group are plotted with the bars representing the median and interquartile range. *** *p* < 0.001.

Bioinformatics analyses of the pooled plasma cfDNA collected at the Pre time point after the NGS run, identified spcfDNA sequences as significant top 5 peaks (Figure 3a). We chose to focus on the top peak of chromosome no. 1 based on the *p*-value and its expression (Figure 3b). This spcfDNA was referred to as spcfDNA-1. TPM and coverage values of the spcfDNA-1 region were approximately 17,600-fold higher than that of the gGAPDH region (Figure 3b,c). Consistent with these results, the median CT values on TaqMan-qPCR assay for the spcfDNA-1 on individual cfDNAs were lower than the median CT value of the gGAPDH fragments (Figure 3d). Additionally, standard deviations (SD) of the individual CT values upon duplicate measurements for the gGAPDH fragments were significantly higher than that of spcfDNA-1 fragments (Figure 3e). Additionally, the spcfDNA-1 fragments in individual cfDNAs were approximately 16,600-fold higher than the gGAPDH fragments as a median (Figure 3f).

### 3.3. spcfDNA-1 Fragments and General Stress Markers Increased with Full Marathon as an Extreme Exercise

**Figure 4:**
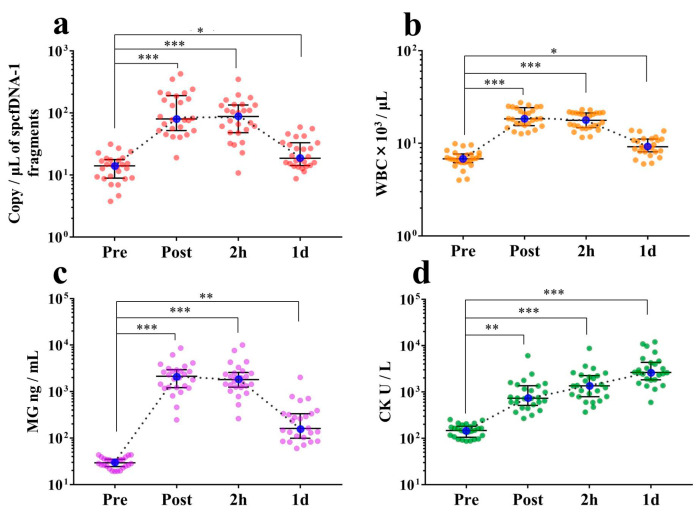
Analyses of the dynamics of the spcfDNA-1 fragments relative to running a full marathon. (**a**) spcfDNA-1 fragments; (**b**) WBCs; (**c**) MG; and (**d**) CK. The individual data of *n* = 26 per group are plotted with the bars representing the median and interquartile range. * *p* < 0.05, ** *p* < 0.01, *** *p* < 0.001. Data regarding these general markers have been previously published online [13].

At the Post and 2 h time points, the levels of spcfDNA-1 fragments were significantly increased (median of 5-fold to 6-fold) compared to the Pre time point. Moreover, the amounts of spcfDNA-1 fragments remained slightly higher (1.3-fold) at the 1 d time point compared to the Pre time point (Figure 4a). WBC and MG values demonstrated similar dynamics to spcfDNA-1 values (Figure 4b,c), with the high values being confirmed at Post and 2 h time points. In contrast, the CK values exhibited different dynamics (Figure 4d), with high values being confirmed at the 1 d time point. Data regarding general markers have been published online in our previous study [13].

### 3.4. Strong Correlations between spcfDNA-1 and General Stress Makers Confirmed

**Figure 5:**
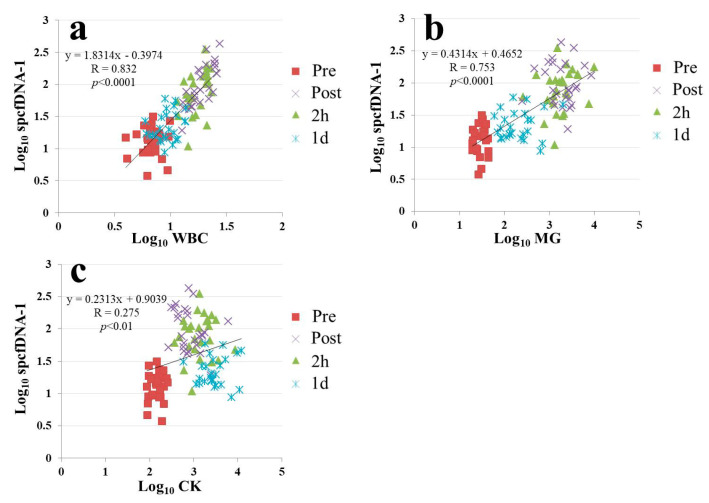
Correlation analyses between spcfDNA-1 and general stress markers. (**a**) WBCs and spcfDNA-1 (**b**) MG and spcfDNA-1 and (**c**) CK and spcfDNA-1.

The spcfDNA-1 values strongly correlated with the WBC or MG values (Figure 5a,b). However, only a weak correlation was observed between the spcfDNA-1 values and CK values (Figure 5c).

## 4. Discussion

In this study, we investigated the presence of spcfDNA fragments in healthy human participants. The results revealed some spcfDNA sequences through NGS and bioinformatics analyses (Figure 3a,b). We subsequently focused on the top spcfDNA fragment and found its amounts were approximately 16,600-fold higher than the cfDNA of the gGAPDH region as normal (Figure 3d,e). This was probably due to the spcfDNA-1 region being degraded much less in the blood than the normal genomic regions. The genome of somatic cells has a chromatin structure containing nucleosomes and many proteins bound to it, such as histones, transcription factors, and co-factors [14,15]. Nucleic acids and nucleosome proteins undergo various modifications for epigenetic regulation, such as acetylation and methylation [16,17,18]. Therefore, depending on the protein(s) binding or the type of chemical modification, regions would vary in their susceptibility to degradation by DNase enzymes. Determining the mode of degradation of the plasma cfDNA in detail was not possible in this study. Therefore, we will investigate the mode of cfDNA degradation using an in vitro model that mimics blood.

It is well known that the concentration of plasma cfDNA that can be extracted is very low, making it difficult to amplify target genome-DNA fragments using qPCR. In general, if the concentration of target DNA fragments is very low, the CT value would be high, and the quantitative ability will be significantly lost because variation would become large. In fact, concentrations of the cfDNA extracted from 200 µL plasma of the individual samples in this study could not be measured using a spectrophotometer because they were very low. In addition, when amplified by targeting a normal genomic region (gGAPDH) in this study, the median CT value was 34.4, and the SDs were significantly high (Figure 3d,e), which means quantitative ability was significantly lost. On the other hand, the median CT value for the spcfDNA-1 fragments in this study was 20.4, a difference of −14 in CT value compared to that of the gGAPDH fragments (Figure 3d). Moreover, the SDs were significantly low (Figure 3e), meaning quantitative ability was not lost. Therefore, quantifying spcfDNA-1 was highly sensitive, and analyzing plasma cfDNA dynamics was quantitatively stable. Moreover, because the spcfDNA-1 target was highly sensitive, it may be possible to analyze the dynamics of spcfDNA-1 fragments under various stresses using a single drop of blood collected from the fingertip. For example, if we can develop a small device that can quantify spcfDNA-1, it would be possible to quantify various stress levels using a drop of blood collected on-site from the fingertip in sports.

In our dynamic analyses, concentrations of the pooled total cfDNA were dramatically increased (almost 10-fold) at the Post time point compared to the Pre time point. The concentrations subsequently returned to baseline at the 2 h time point (Figure 2a,b). In contrast, spcfDNA-1 fragments were significantly increased (approximately 6-fold) at both the Post and 2 h time points compared to the Pre time point. Even after 1 d, there was a significant increase in spcfDNA-1 fragments (Figure 4a). Therefore, regarding the full marathon, we considered the spcfDNA-1 fragments better reflectors of prolonged stress than the total cfDNA.

In dynamic analyses including general stress makers, the spcfDNA-1 fragments showed similar dynamics to WBCs and MG, with high values being confirmed at the Post and 2 h time points and then returning to near baseline at 1 d (Figure 4a–c). Meanwhile, CK demonstrated differential dynamics, with the highest values being observed at 1 d (Figure 4d). MG is known to leak into the blood immediately after muscle damage, and WBCs reflect an acute inflammatory response. Previous reports have also shown that WBC and MG levels in the blood are significantly increased immediately after running a marathon race [19,20,21]. In addition, strong correlations were confirmed in our current study between spcfDNA-1 and WBC or MG (Figure 5a,b). On the other hand, although a significant correlation was observed between CK and spcfDNA-1, the correlation was not strong (Figure 5c). Therefore, spcfDNA-1 may serve as a new sensitive biomarker for quantifying acute physical stress in sports research.

This study has some limitations. The aerobic capacities of the participants were not evaluated in this study, meaning physical stress may not be unified among the individuals. Therefore, further additional research is needed, such as changing exercise intensity and sports type, to establish robust scientific evidence for spcfDNA-1 as a new biomarker. It is also considered very important to carry out experiments in which exercise intensity is accurately controlled, using indicators such as maximal oxygen uptake (VO_2_ max), lactate threshold (LT), and ventilation threshold (VT). After additional research, if robust scientific evidence is accumulated, spcfDNA-1 can be targeted in exercise physiology researchers as a physical stress biomarker in any type of sports or exercise.

## 5. Conclusions

We first identified spcfDNA sequences called spcfDNA-1 present in the plasma at 16,600-times higher levels than that in a normal genomic region and was highly sensitive to the TaqMan-qPCR assay. We also found that the levels of the spcfDNA-1 fragments fluctuated significantly upon extreme exercise and severe physiological stress, such as that caused by running a full marathon. The spcfDNA-1 fragment levels strongly correlated with WBC and MG levels. Overall, the findings of this study provide new insights into exercise physiology and genome biology in humans.

## Figures and Tables

**Table 1:** Sequences of the PCR primers and TaqMan probes used in this study.

Targets		Sequences (5′ to 3′)	Predicted Amplicon size (bp)
gGAPDH	Forward	GCTCTTAAAAAGTGCAGGGTCTG	154
	Probe	56-FAM/CTTCTAGGT/ZEN/ATGACAACGAATTTGG/3IABkFQ	
	Reverse	GGTCTTACTCCTTGGAGGCCA	
spcfDNA-1	Forward	TCTTGTGGCCTTCGTTGGAA	181
	Probe	56-FAM/ATTGACCTC/ZEN/AAAGCGGCTGA/3IABkFQ	
	Reverse	ATTGACCTCAAAGCGGCTGA	

gGAPDH: genomic GAPDH; spcfDNA-1: spcfDNA sequences.

## Data Availability

The NGS data presented in this study are not available because it include personal information.
